# Wada test results contribute to the prediction of change in verbal learning and verbal memory function after temporal lobe epilepsy surgery

**DOI:** 10.1038/s41598-021-90376-3

**Published:** 2021-05-26

**Authors:** Nadine Conradi, Friederike Rosenberg, Susanne Knake, Louise Biermann, Anja Haag, Iris Gorny, Anke Hermsen, Viola von Podewils, Marion Behrens, Marianna Gurschi, Richard du Mesnil de Rochemont, Katja Menzler, Sebastian Bauer, Susanne Schubert-Bast, Christopher Nimsky, Jürgen Konczalla, Felix Rosenow, Adam Strzelczyk

**Affiliations:** 1 Department of Neurology, Epilepsy Center Frankfurt Rhine-Main, University Hospital Frankfurt and Goethe University, Schleusenweg 2-16, 60528 Frankfurt am Main, Germany; 2 LOEWE Center for Personalized Translational Epilepsy Research (CePTER), Goethe University, Frankfurt am Main, Germany; 3 Department of Neurology, Epilepsy Center Hessen, Philipps-University Marburg, Marburg, Germany; 4 Department of Neurology, University Medicine Greifswald, Greifswald, Germany; 5 Department of Neurology, University Hospital Frankfurt and Goethe University, Frankfurt am Main, Germany; 6 Department of Neuroradiology, Philipps-University Marburg, Marburg, Germany; 7 Institute of Neuroradiology, University Hospital Frankfurt and Goethe University, Frankfurt am Main, Germany; 8 Department of Neuropediatrics, University Hospital Frankfurt and Goethe University, Frankfurt am Main, Germany; 9 Department of Neurosurgery, Philipps-University Marburg, Marburg, Germany; 10 Department of Neurosurgery, University Hospital Frankfurt and Goethe University, Frankfurt am Main, Germany

**Keywords:** Epilepsy, Psychology, Neurology

## Abstract

In recent years, the clinical usefulness of the Wada test (WT) has been debated among researchers in the field. Therefore, we aimed to assess its contribution to the prediction of change in verbal learning and verbal memory function after epilepsy surgery. Data from 56 patients with temporal lobe epilepsy who underwent WT and subsequent surgery were analyzed retrospectively. Additionally, a standard neuropsychological assessment evaluating attentional, learning and memory, visuospatial, language, and executive function was performed both before and 12 months after surgery. Hierarchical linear regression analyses were used to determine the incremental value of WT results over socio-demographic, clinical, and neuropsychological characteristics in predicting postsurgical change in patients’ verbal learning and verbal memory function. The incorporation of WT results significantly improved the prediction models of postsurgical change in verbal learning (*∆R*^2^ = 0.233, *p* = .032) and verbal memory function (*∆R*^2^ = 0.386, *p* = .005). Presurgical performance and WT scores accounted for 41.8% of the variance in postsurgical change in verbal learning function, and 51.1% of the variance in postsurgical change in verbal memory function. Our findings confirm that WT results are of significant incremental value for the prediction of postsurgical change in verbal learning and verbal memory function. Thus, the WT contributes to determining the risks of epilepsy surgery and, therefore, remains an important part of the presurgical work-up of selected patients with clear clinical indications.

## Introduction

The Wada test (WT), introduced in 1949 by Juhn Atsushi Wada as a procedure to determine hemispheric language lateralization (HLL)^1^, was further developed in 1962 to additionally assess hemispheric memory lateralization (HML)^2^. Because the WT allows the imitation of potential effects of epilepsy surgery through the temporary inactivation of one hemisphere, this procedure evolved as the gold standard to (1) evaluate the functional adequacy of the hemisphere ipsilateral to the epileptogenic focus (epileptogenic hemisphere), and (2) assess the functional reserve capacity of the hemisphere contralateral to the epileptogenic focus (non-epileptogenic hemisphere)^3,4^. The WT became an important part of the presurgical work-up of epilepsy surgery candidates and was applied, together with comprehensive neuropsychological assessments, to provide accurate predictions of a patient’s possible postsurgical cognitive impairment^5,6^.

Given the well-documented interdependence between cognitive decline and reduced quality of life after epilepsy surgery^7–10^, the presurgical prediction of potential cognitive risks by determining the patients’ hemispheric language and memory lateralization is essential for balancing out the risks and benefits of epilepsy surgery on an individual level. Particularly in patients with temporal lobe epilepsy (TLE), the effects of surgery on cognitive functioning have been comprehensively investigated^11,12^. Approximately 30–40% of TLE patients experience postsurgical decline in memory function, with the highest decline rates seen after surgeries within the language-dominant hemisphere^13,14^. Using multivariate approaches, several predictor variables for change in memory function after epilepsy surgery have been identified. A study not including WT results^15^ described the presence of focal to bilateral tonic–clonic seizures before surgery, the side of surgery (left/right hemisphere), the inclusion of the hippocampus in the resection, and the patients’ presurgical cognitive performance to be significantly predictive for postsurgical change in verbal memory function. Another study not involving WT results^16^ found the patients’ age, age at onset of epilepsy, the etiology of epilepsy, the side of surgery (left/right hemisphere), and the patients’ presurgical cognitive performance to be significant predictors for memory decline after temporal lobe surgery.

The value of *WT results* for the prediction of postsurgical change in memory function remains controversial. On the one hand, a number of studies indicated the usefulness of WT results for this purpose. For example, Cohen-Gadol et al.^17^ provided evidence for the interpretation of WT scores as indicators of the functional integrity of the patients’ hippocampi. Furthermore, in a multivariate prediction model^18^, WT scores, together with the side of surgery (language-dominant/non-dominant hemisphere), the patients’ presurgical cognitive performance, and the presence of hippocampal sclerosis as the etiology of epilepsy, were identified as significant predictor variables for postsurgical verbal memory decline. Another study^19^ described WT scores, together with the patients’ age, age at onset of epilepsy, and presurgical cognitive performance, to be significantly predictive for verbal memory decline after temporal lobe epilepsy surgery. While some studies identified WT scores evaluating the functional adequacy of the epileptogenic hemisphere^18,20^ to be more helpful, other studies described WT scores assessing the functional reserve capacity of the non-epileptogenic hemisphere^21,22^ to be more useful; and yet others concluded that the combination of both WT scores^17,23^ was most suitable for the prediction of possible postsurgical cognitive impairment.

On the other hand, a variety of studies exist^24–27^ that doubt the significance of WT results for the prediction of postsurgical memory decline. For example, Elshorst et al.^28^ concluded that WT scores were of no added value for the prediction of postsurgical change in verbal memory function and identified the etiology of epilepsy and the patients’ presurgical cognitive performance to be the only significant predictor variables in their multivariate model. Additionally, reports of false-positive WT results (i.e., falsely predicting postsurgical memory impairment)^29^ and limited reproducibility of Wada HML results^30^ advanced skepticism towards the predictive value of WT results.

With the development of non-invasive alternatives, the use of the WT has decreased in recent years^31–33^. According to a large survey^34^, in 91% of epilepsy centers across Europe, functional MRI (fMRI) is the current standard method for the assessment of HLL, which has been demonstrated to be a reliable substitute for WT^35^. Some centers also use functional transcranial Doppler sonography (fTCD), transcranial magnetic stimulation (TMS), or magnetoencephalography (MEG) to assess HLL. HML is typically assessed through elaborate neuropsychological assessments, though a number of studies have investigated the use of verbal memory paradigms in fMRI for this purpose, as summarized by Massot-Tarrús et al.^36^.

As researchers and clinicians increasingly question the remaining usefulness of the WT for the presurgical assessment of epilepsy surgery candidates, this study was designed to contribute to the answer of this continuously debated issue. Building upon the results of the large variety of earlier studies, we followed a multivariate approach and aimed to determine the *incremental* value of WT results over socio-demographic, clinical, and neuropsychological characteristics in predicting change in patients’ verbal learning and verbal memory function after temporal lobe epilepsy surgery.

## Materials and methods

### Patients

This retrospective study collected longitudinal data from a consecutive clinical sample of 69 TLE patients who received technically valid bilateral WT and underwent epilepsy surgery at the Epilepsy Center Hessen (Marburg, Germany) or the Epilepsy Center Frankfurt Rhine-Main (Frankfurt am Main, Germany) between 2000 and 2019. Additionally, a standard neuropsychological test battery^37–39^ was performed both before and 12 months after surgery. Patients with incomplete neuropsychological data due to language barriers (*n* = 7) or intellectual disabilities (*n* = 6) were excluded (18.8%), resulting in a final sample of 56 TLE patients (55.4% men; median age = 38.0 years, range = 12–59 years).

WT was performed as part of the presurgical work-up to determine hemispheric language and memory lateralization when non-invasive imaging techniques (i.e., repeated fTCD and/or fMRI) failed to provide conclusive HLL results (e.g., due to technical difficulties, insufficient temporal bone windows, or the patient’s inadequate task performance).

The study was approved by the Ethics Committee of the University of Frankfurt Medical Faculty. All methods were performed in accordance with the relevant guidelines and regulations defined by the Declaration of Helsinki. Informed consent was waived by the ethics committee due to the retrospective nature of the analysis.

Epilepsy syndrome diagnoses were obtained during video-EGG-monitoring, and the classifications of epilepsies and etiologies were based on the definitions proposed by the ILAE^40–43^ and the four-dimensional epilepsy classification^44–46^. Seizure outcome was classified using the Engel Epilepsy Surgery Outcome Scale^47^.

### Wada testing procedure

As described elsewhere^48^, all patients underwent internal carotid artery catheterization via the right femoral artery. After a contrast medium was injected to determine the location of the catheter and to control for crossflow, a barbiturate injection (using amobarbital in 75.0% and methohexital in 25.0% of patients) followed, leading to an inactivation of brain structures supplied by the injected vessel. All patients were monitored with simultaneous EEG recording during WT. Once effective hemispheric inactivation was confirmed by EEG slowing and a contralateral grip strength of 0/5, testing began.

Language testing included spontaneous speech (10 points), speech comprehension (5 points), sentence repetition (2 points), object naming (4 points), and word reading (8 points). For each procedure, a Wada language score (range = 0–29) reflecting the language function of the non-inactivated hemisphere was computed. A Wada language laterality index was determined by subtracting the Wada language score representing left-sided language function (obtained after inactivation of the right hemisphere) from the Wada language score representing right-sided language function (obtained after inactivation of the left hemisphere). The Wada language laterality index could range between − 29 (representing complete left-sided HLL) and + 29 (representing complete right-sided HLL).

Memory testing included the delayed recognition of object sketches (4 points), portrait photographs (4 points), words (4 points), and abstract words (4 points) that had been visually presented during language testing 15–30 min prior. For each procedure, the proportion of correctly recognized items was computed as follows: (recognized items ÷ 16) × 100. The resulting Wada memory score (range = 0–100%) reflected the memory function of the non-inactivated hemisphere. A Wada memory laterality index, computed analogously to the Wada language laterality index, could range between − 100 (representing complete left-sided HML) and + 100 (representing complete right-sided HML).

The epileptogenic hemisphere was injected first. After the procedure was completed, the process was repeated in the non-epileptogenic hemisphere. Wada language scores representing language function of the epileptogenic hemisphere (obtained after inactivation of the non-epileptogenic hemisphere) are referred to as “ipsilateral Wada language scores”, whereas Wada language scores representing language function of the non-epileptogenic hemisphere (obtained after inactivation of the epileptogenic hemisphere) are termed “contralateral Wada language scores”. The same applies for Wada memory scores.

### Neuropsychological assessment

The standard neuropsychological test battery evaluating attentional, learning and memory, visuospatial, language, and executive function was administered at the presurgical (baseline scores) and postsurgical (retest scores) assessment, as described elsewhere^10,37–39^. Intelligence (verbal IQ) was measured using the Wechsler Intelligenztest für Erwachsene (WIE)^49^ in 78.6% of patients, and a multiple-choice vocabulary test (Mehrfachwahl-Wortschatz-Intelligenztest)^50^ in 21.4% of patients. Handedness was determined by the Edinburgh Handedness Inventory (EHI)^51^. Trained neuropsychologists performed all neuropsychological assessments in a standardized manner. Patients were confirmed as not currently being treated with topiramate or benzodiazepines and not having seizures or status epilepticus within the 24 h before or during the assessments.

### Outcome measures

The Verbaler Lern- und Merkfähigkeitstest (VLMT)^52^ was used to assess patients’ verbal learning and verbal memory function. In the VLMT, a patient must learn a list of 15 words within five repetitions (VLMT-1 = first repetition, learned words; VLMT-5 = fifth repetition, learned words; VLMT-Sum = all repetitions, sum of learned words). Then, a distraction list must be learned (VLMT-B = distraction list, learned words), and the words of the initial list must be recalled afterwards (VLMT-56 = recall after distraction, forgotten words). Later, the words must be recalled after a 30-min delay (VLMT-7 = delayed recall; VLMT-57 = delayed recall, forgotten words). Finally, the words must be recognized out of fifty words (VLMT-R = recognition; VLMT-RM = recognition minus mistakes).

Age-adjusted norms from test manuals were used to calculate *z*-scores for baseline- and retest VLMT test scores. For the pre- and postsurgical assessment, verbal learning function was defined as the median of VLMT-1, VLMT-5, VLMT-Sum, and VLMT-B *z*-scores, while verbal memory function was defined as the median of VLMT-56, VLMT-7, VLMT-57, VLMT-R, and VLMT-RM *z*-scores for each patient. Following that, baseline median *z*-scores were subtracted from the respective retest median *z*-scores to determine the two outcome measures: (a) postsurgical change in verbal learning function, and (b) postsurgical change in verbal memory function.

### Potential predictor variables

Based on clinical considerations, feasibility, and previous findings, three groups of potential predictor variables for postsurgical change in verbal learning and verbal memory function were defined:

1. *Socio-demographic and clinical characteristics* including the patients’ age, age at onset of epilepsy in years^16,19^, and the presence or absence of hippocampal sclerosis as the etiology of epilepsy^15,16,18,28^.
2. *Neuropsychological variables* including the patients’ presurgical cognitive performance, as assessed by baseline verbal learning and verbal memory function^15,16,18,19,28^.
3. *Wada test scores and results* including ipsilateral and contralateral Wada language and memory scores^18,19^, and side of surgery, as assessed by Wada HLL (language-dominant or non-dominant hemisphere) and HML results (memory-dominant or non-dominant hemisphere)^15,16,18^.

### Statistical analyses

Descriptive analyses were conducted for the socio-demographic and clinical characteristics of the sample, and the WT scores and results regarding hemispheric language and memory lateralization. The patients’ verbal learning and verbal memory function were compared between the pre- and postsurgical assessment using Wilcoxon tests for the median test scores and McNemar’s tests for the classification as a relevant deficit (i.e., *z*-score < − 1.0). Missing values for each test score were replaced by the median test score in the sample.

After computing collinearity statistics, hierarchical linear regression analyses were performed, and the groups of potential predictor variables were entered into the regression models in three blocks to determine the incremental value of (3) *Wada test scores and results* over (1) *Socio-demographic and clinical characteristics*, and (2) *Neuropsychological variables* in predicting (a) postsurgical change in verbal learning function, and (b) postsurgical change in verbal memory function. Identical statistical procedures were performed for both outcome measures, and analyses were carried out using IBM SPSS Statistics 22 (IBM Corporation, Armonk, New York, USA).

## Results

The socio-demographic and clinical characteristics of the sample are summarized in Table 1. Twelve months after epilepsy surgery, 40 patients were free of disabling seizures (71.4%, Engel class I), among which 31 patients were completely seizure-free since surgery (55.4%, Engel class IA). Six patients had rare disabling seizures (10.7%, Engel class II), six patients achieved a worthwhile improvement (10.7%, Engel class III), and four patients experienced no worthwhile improvement in seizure control (7.1%, Engel class IV).

**Table 1:** Socio-demographic and clinical characteristics of the sample (*n* = 56).

	Number (%)
**Gender**	
Female	25 (44.6)
Male	31 (55.4)
**Handedness**	
Consistently right-handed (EHI ≥ 50)	45 (80.4)
Consistently left-handed (EHI ≤ − 50)	6 (10.7)
Ambidextrous	5 (8.9)
**Education**	
≤ 9 years (German Hauptschulabschluss)	26 (46.4)
10–12 years (German Realschulabschluss)	22 (39.3)
> 12 years (German Abitur)	8 (14.3)
**Lateralization of TLE**	
Left-sided focus	40 (71.4)
Right-sided focus	16 (28.6)
**Etiology**	
Hippocampal sclerosis	33 (58.9)
Arteriovenous malformation	6 (10.7)
Low grade epilepsy-associated tumor	8 (14.3)
Focal cortical dysplasia	2 (3.6)
Unknown	7 (12.5)
**Surgical procedure**	
Classical two-third temporal lobectomy	8 (14.3)
Amygdalohippocampectomy incl. temporal pole	9 (16.1)
Selective amygdalohippocampectomy	28 (50.0)
Extended lesionectomy	11 (19.6)
**Seizure outcome after surgery**	
Engel class I (free of disabling seizures)	40 (71.4)
Engel class II (rare disabling seizures)	6 (10.7)
Engel class III (worthwhile improvement)	6 (10.7)
Engel class IV (no worthwhile improvement)	4 (7.1)

	Median (range)
Age (years)	38.0 (12 to 59)
Age at onset of epilepsy (years)	16.0 (1 to 38)
Age at surgery (years)	37.5 (12 to 60)
Intelligence (verbal IQ)	92.0 (74 to 128)

A summary of WT scores and results regarding hemispheric language and memory lateralization is presented in Table 2. Hemispheric language lateralization was left-sided in 47 patients (83.9%), right-sided in five patients (8.9%), and bilateral in four patients (7.1%). Hemispheric memory lateralization was left-sided in 21 patients (37.5%), right-sided in 18 patients (32.1%), and bilateral in 17 patients (30.4%). No medical complications (such as clinical or EEG seizures, strokes, or allergic reactions to the contrast medium) were observed during WT.

**Table 2:** Summary of Wada test scores and results regarding hemispheric language and memory lateralization.

	Number (%)
**Hemispheric language lateralization**	
Language left-sided	47 (83.9)
Language right-sided	5 (8.9)
Language bilateral	4 (7.1)
**Hemispheric memory lateralization**	
Memory left-sided	21 (37.5)
Memory right-sided	18 (32.1)
Memory bilateral	17 (30.4)

	Median (range)
Ipsilateral Wada language scores	23.0 (0 to 29)
Contralateral Wada language scores	12.0 (0 to 29)
Wada language laterality indices (range = − 29 to 29)	− 16.0 (− 29 to 23)
Ipsilateral Wada memory scores	38.0 (6 to 88)
Contralateral Wada memory scores	56.0 (13 to 88)
Wada memory laterality indices (range = − 100 to 100)	0.0 (− 63 to 62)

The patients’ verbal learning and verbal memory function at the pre- and postsurgical assessment are depicted in Table 3. A significant decline in patients’ median verbal memory function was observed between the pre- and postsurgical assessment (*p* = 0.003), while no significant change in patients’ median verbal learning function was found. The proportion of patients classified as showing relevant deficits significantly increased between the pre- and postsurgical assessment for verbal learning function (39.3% vs. 53.6%, *p* = 0.039) and verbal memory function (44.6% vs. 67.9%, *p* = 0.002).

**Table 3:** Verbal learning and verbal memory function at the pre- and postsurgical assessment.

Test score	Baseline scores		Retest scores	
	Median (range)		Median (range)	
	Raw scores	*z*-scores	Raw scores	*z*-scores
**Verbal learning function**				
VLMT first repetition, LW	6.0 (3 to 10)	− 0.52 (− 1.75 to 1.75)	6.0 (1 to 12)	− 0.52 (− 1.75 to 1.75)
VLMT fifth repetition, LW	11.0 (4 to 15)	− 1.04 (− 1.75 to 1.65)	11.0 (3 to 15)	− 1.16 (− 1.75 to 0.84)
VLMT all repetitions, sum of LW	45.5 (22 to 68)	− 0.84 (− 1.75 to 1.75)	41.5 (16 to 72)	− 1.04 (− 1.75 to 1.75)
VLMT distraction list, LW	5.0 (2 to 10)	− 0.84 (− 1.75 to 1.75)	5.0 (1 to 11)	− 1.04 (− 1.75 to 1.75)
= ^a^median verbal learning function		− 0.82 (− 1.75 to 1.46)		− 1.05 (− 1.75 to 1.75)
**Verbal memory function**				
VLMT recall after distraction, FW	2.0 (− 5 to 7)	− 0.52 (− 1.75 to 1.75)	3.0 (− 4 to 11)	− 1.28 (− 1.75 to 1.75)
VLMT delayed recall	8.0 (1 to 15)	− 1.28 (− 1.75 to 1.28)	6.0 (0 to 15)	− 1.75 (− 1.75 to 1.28)
VLMT delayed recall, FW	2.0 (− 7 to 6)	− 0.67 (− 1.75 to 1.75)	2.0 (− 7 to 8)	− 0.67 (− 1.75 to 1.75)
VLMT recognition	14.0 (8 to 15)	− 0.76 (− 1.75 to 0.67)	12.0 (6 to 15)	− 1.04 (− 1.75 to 0.67)
VLMT recognition, mistakes	2.0 (0 to 16)	− 0.94 (− 1.75 to 0.67)	3.0 (0 to 16)	− 1.28 (− 1.75 to 0.84)
= ^a^median verbal memory function**		− 0.67 (− 1.75 to 0.67)		− 1.28 (− 1.75 to 1.28)

LW, learned words; FW, forgotten words.

^a^Median test scores compared between the two assessments using Wilcoxon tests, **p < .01.

### Hierarchical linear regression analyses

No signs of multicollinearity between predictor variables were found.

As depicted in Table 4, socio-demographic and clinical characteristics, entered as predictor variables in the first block of the analysis, did not account for a significant amount of variance in postsurgical change in verbal learning function (*model* 1: 5.5%, *p* = 0.454). Adding neuropsychological predictor variables in the second block led to a significant increase in the proportion of explained variance (*p* = 0.010); however, *model* 2 still did not account for a significant amount of variance in the outcome variable (18.5%, *p* = 0.051). WT scores and results, added as predictor variables in the third block, accounted for a further significant increase in the proportion of explained variance (*p* = 0.032), which led to a significant *model* 3, explaining 41.8% of the variance in the outcome variable (*p* = 0.010). Examination of individual predictor variables indicated that lower baseline verbal learning scores (*β* = − 0.60, *p* = 0.005), lower ipsilateral Wada language scores (*β* = − 1.09, *p* = 0.032), and higher contralateral Wada language scores (*β* = 0.72, *p* = 0.025) were significantly predictive for a better postsurgical outcome in verbal learning function.

**Table 4:** Results of the hierarchical linear regression analysis for the prediction of postsurgical change in verbal learning function.

Predictor variables	*B*	*SE*	*β*	*R*^2^	*∆R*^2^
**Model 1**				0.055	
Age^1^	− 0.00	0.01	− 0.14		
Age at onset of epilepsy^1^	− 0.01	0.01	− 0.18		
Etiology of hippocampal sclerosis^1^	0.26	0.22	0.31		
**Model 2**				0.185	0.130*
Age^1^	− 0.01	0.01	− 0.32		
Age at onset of epilepsy^1^	− 0.01	0.01	− 0.28		
Etiology of hippocampal sclerosis^1^	0.16	0.21	0.19		
Baseline verbal learning function^2^	− 0.31	0.12	− 0.51*		
**Model 3**				0.418*	0.233*
Age^1^	− 0.01	0.01	− 0.71		
Age at onset of epilepsy^1^	− 0.00	0.01	− 0.07		
Etiology of hippocampal sclerosis^1^	0.28	0.21	0.33		
Baseline verbal learning function^2^	− 0.36	0.12	− 0.60**		
Ipsilateral Wada language scores^3^	− 0.03	0.01	− 1.09*		
Contralateral Wada language scores^3^	0.03	0.01	0.72*		
Wada HLL results^3^	0.50	0.40	0.63		
Ipsilateral Wada memory scores^3^	− 0.00	0.01	− 0.19		
Contralateral Wada memory scores^3^	0.00	0.01	0.08		
Wada HML results^3^	0.16	0.30	0.11		

Predictor variables entered the regression models in the first^1^, second^2^, and third^3^ block.

*B*, unstandardized coefficient; *SE*, standard error; *β*, standardized coefficient; HLL, hemispheric language lateralization; HML, hemispheric memory lateralization, *p < .05; **p < .01.

Etiology of hippocampal sclerosis categorized as 1 = present; 0 = absent. Wada HLL results categorized as 1 = surgery within the language-dominant hemisphere; 0 = surgery within the non-dominant hemisphere. Wada HML results categorized as 1 = surgery within the memory-dominant hemisphere; 0 = surgery within the non-dominant hemisphere.

As shown in Table 5, socio-demographic and clinical characteristics, entered as predictor variables in the first block of the analysis, did not account for a significant amount of variance in postsurgical change in verbal memory function (*model* 1: 12.2%, *p* = 0.108). Neuropsychological variables, added as predictor variables in the second block, accounted for a significant increase in the proportion of explained variance (*p* = 0.018), which resulted in a significant *model* 2, accounting for 22.6% of the variance in the outcome variable (*p* = 0.019). Evaluation of individual predictor variables revealed that baseline verbal memory scores (*β* = − 0.56, *p* = 0.018) were significantly predictive for postsurgical change in verbal memory function. Adding WT scores and results in the third block of the analysis led to a further significant increase in the proportion of explained variance (*p* = 0.005), which produced a significant *model* 3, explaining 51.1% of the variance in the outcome variable (*p* = 0.001). Examination of individual predictor variables indicated that lower baseline verbal memory scores (*β* = − 0.93, *p* < 0.001), higher ipsilateral Wada memory scores (*β* = 0.92, *p* = 0.049), and Wada HLL results indicating surgery within the language-non-dominant hemisphere (*β* = − 1.20, *p* = 0.013) were significantly predictive for a better postsurgical outcome in verbal memory function.

**Table 5:** Results of the hierarchical linear regression analysis for the prediction of postsurgical change in verbal memory function.

Predictor variables	*B*	*SE*	*β*	*R*^2^	*∆R*^2^
**Model 1**				0.122	
Age^1^	− 0.01	0.01	− 0.72		
Age at onset of epilepsy^1^	0.01	0.01	0.23		
Etiology of hippocampal sclerosis^1^	0.23	0.22	0.27		
**Model 2**				0.226*	0.103*
Age^1^	− 0.01	0.01	− 0.67		
Age at onset of epilepsy^1^	− 0.00	0.01	− 0.04		
Etiology of hippocampal sclerosis^1^	− 0.02	0.23	− 0.03		
Baseline verbal memory function^2^	− 0.34	0.14	− 0.56*		
**Model 3**				0.511**	0.285**
Age^1^	− 0.00	0.01	− 0.26		
Age at onset of epilepsy^1^	0.01	0.01	0.31		
Etiology of hippocampal sclerosis^1^	− 0.00	0.21	− 0.00		
Baseline verbal memory function^2^	− 0.57	0.13	− 0.93***		
Ipsilateral Wada language scores^3^	− 0.01	0.01	− 0.33		
Contralateral Wada language scores^3^	− 0.01	0.01	− 0.24		
Wada HLL results^3^	− 0.97	0.37	− 1.20*		
Ipsilateral Wada memory scores^3^	0.01	0.01	0.92*		
Contralateral Wada memory scores^3^	− 0.00	0.01	− 0.35		
Wada HML results^3^	− 0.20	0.27	− 0.14		

Predictor variables entered the regression models in the first^1^, second^2^, and third^3^ block.

*B*, unstandardized coefficient; *SE*, standard error; *β*, standardized coefficient; HLL, hemispheric language lateralization; HML, hemispheric memory lateralization, *p < .05; **p < .01; ***p < .001.

Etiology of hippocampal sclerosis categorized as 1 = present; 0 = absent. Wada HLL results categorized as 1 = surgery within the language-dominant hemisphere; 0 = surgery within the non-dominant hemisphere. Wada HML results categorized as 1 = surgery within the memory-dominant hemisphere; 0 = surgery within the non-dominant hemisphere.

## Discussion

Although the Wada test (WT) was initially established as the gold standard for determining hemispheric language and memory lateralization, its application in the presurgical work-up of epilepsy patients has decreased in recent years^31–33^. The literature lacks consensus regarding the predictive value of WT results for possible postsurgical cognitive impairment, and researchers in the field have continuously discussed the remaining contribution of the WT to determining the risks of epilepsy surgery for individual patients^24–27^. Therefore, this study was designed to help clarify this issue by building upon the results of the large number of previous studies that identified several variables potentially predicting change in memory function after epilepsy surgery^15,16,28^ and indicated the usefulness of WT results for this purpose^17–19^. Following a multivariate approach, we aimed to determine the *incremental* value of WT results over socio-demographic, clinical, and neuropsychological characteristics in predicting change in patients’ verbal learning and verbal memory function after temporal lobe epilepsy surgery.

In line with previous results^11–14^, we observed a significant increase in the proportion of TLE patients showing relevant deficits in verbal learning and verbal memory function after surgery. Furthermore, our results confirmed earlier findings^15,16,18,19,28^ indicating lower presurgical cognitive performance to be predictive of a better postsurgical outcome in verbal learning and verbal memory function. However, in our sample, no significant predictive value could be identified for the patients’ age, age at onset of epilepsy, or the presence of hippocampal sclerosis as the etiology of epilepsy, as suggested by these studies.

Regarding HLL, our WT results corresponded well with the frequency distribution of HLL described in the literature^53^. In accordance with previous findings^18,19^, lower ipsilateral Wada language scores (i.e., reduced functional adequacy of the epileptogenic hemisphere) and higher contralateral Wada language scores (i.e., increased functional reserve capacity of the non-epileptogenic hemisphere) were predictive for a better postsurgical outcome in verbal learning function. This result supports the conclusion of earlier studies^17,23^, suggesting a combination of ipsilateral and contralateral WT scores to be most suitable for the prediction of postsurgical cognitive change. Moreover, we found the side of surgery, as assessed by WT results indicating HLL within the non-epileptogenic hemisphere (i.e., resulting in surgery within the language-non-dominant hemisphere) to be predictive for a better postsurgical outcome in verbal memory function, as described previously^10,13–16,18^.

By contrast, our WT results regarding HML were distributed rather surprisingly (see Table 2) and did not correspond well to our Wada HLL results (located within the same hemisphere in only 42.9% of patients). Also, unexpectedly, higher ipsilateral Wada memory scores were predictive of a better postsurgical outcome in verbal memory function. These counterintuitive findings raise questions regarding the ability of the WT to appropriately assess HML. However, given our knowledge regarding the material-specificity of memory functions^54,55^, the selection of stimulus material for memory testing during WT might provide an explanation for our findings. In this study, memory testing included a mixture of visually presented verbal (words and abstract words) and non-verbal (object sketches and portrait photographs) stimuli, yet, they were summarized into one general Wada memory score representing the proportion of correctly recognized items of both groups. Thus, our WT procedure may have not only assessed verbal HML (found to be especially related to the language-dominant hemisphere) but also (if not exclusively) measured non-verbal HML (found to be particularly related to the language-non-dominant hemisphere)^54,55^. Therefore, according to this explanation, the equal distribution of Wada HML results to the left and right hemisphere we obtained appears to be plausible.

Most importantly, in contrast to studies^24–26,28–30^ describing WT results to be of no added value for the prediction of postsurgical cognitive impairment, our findings indicate the opposite: The incorporation of WT results significantly improved the prediction models of postsurgical change in verbal learning and verbal memory function in our sample. While these results initially seem contradictory and inconsistent, a more thorough examination of previous studies reveals possible explanations. Overall, the comparability of the variety of studies that investigated the predictive value of WT results is limited due to substantial differences in WT procedures (e.g., regarding the applied barbiturate, the language testing paradigm, or the stimulus material used for memory testing), neuropsychological assessments, the determination of outcome measures, and the choice of potential predictor variables included^24,56^. For example, although Elshorst et al.^28^ pursued a similar multivariate approach as this study, they only included Wada memory scores but not language scores, and entirely omitted WT lateralization results. However, in our study, Wada language scores in particular, as well as Wada HLL results, were identified to be significantly predictive. Thus, taking these factors into consideration, the diverse findings (i.e., not obtaining/obtaining a significant predictive value of WT results) are comprehensible.

### Limitations and future perspectives

Several limitations of the current study deserve further discussion. First, our findings should be interpreted against the retrospective background of this study. Our sample was highly selected, comprised of TLE patients with mainly left-sided epileptogenic focus who underwent WT and subsequent surgery, which might bias the results. Because only patients with inconclusive HLL results from non-invasive imaging techniques (i.e., repeated fTCD and/or fMRI) were included, no analyses could be conducted concerning the additional predictive value of WT results over fMRI findings regarding patients’ hemispheric language and memory lateralization. Furthermore, the influence of the surgical approach on patients’ neuropsychological outcome after surgery has been previously described^57^; accordingly, the heterogeneity of the surgical procedures applied in our sample might limit the generalizability of our results. Lastly, our small sample size relative to the number of analyzed predictor variables implies the risk of potentially overfitting the regression models. However, due to ethical considerations, it would not be possible to enhance the sample size by performing the WT in patients without clinical indications. Moreover, the current study design provides important insights into genuine experiences with the WT in the presurgical work-up of epilepsy patients in clinical routine.

Second, by summarizing verbal and non-verbal stimuli into one general Wada memory score for each hemisphere, no differentiated consideration of material-specific HML was possible. Therefore, in the future, the computation of two separate Wada memory scores for each hemisphere should be integrated into the WT procedure. Further, the development of a commonly accepted standard protocol should be promoted to allow a comprehensive evaluation of verbal and non-verbal HML and to enhance the comparability of WT results from different epilepsy centers.

Third, given the mixture of verbal and non-verbal stimuli used in memory testing during WT, in clinical routine and future studies, WT results should not only be used to predict postsurgical change in verbal but also non-verbal learning and memory function. By extending the outcome measures, and adapting them to the stimulus material used to assess HML during WT, the significance of WT results for the prediction of possible postsurgical cognitive impairment might be considered much more promising in the future.

In general, it appears that the majority of skepticism towards the predictive value of WT results derives from doubts concerning the ability of the WT to appropriately assess HML, as demonstrated by expert ratings in a large multicenter review^32^. In line with this impression, a systematic review^56^ showed that Wada *language* scores rather than Wada memory scores were significantly more sensitive for the prediction of postsurgical verbal memory decline. This finding corresponds to our results and is also reflected by the indication for WT most frequently reported in a large survey of epilepsy centers across Europe^34^ (i.e., the determination of the patients’ hemispheric language lateralization, not memory lateralization).

In accord with previous research^24–26^, we would not recommend the routine performance of WT as part of the presurgical work-up for *all* patients considered as candidates for epilepsy surgery, or to base the decision for or against epilepsy surgery exclusively on WT results. However, as personalized medicine gains increasing importance in clinical routine^58^, the WT might be an instrument to be used in a highly selected subgroup of patients with clear clinical indications. For example, patients with neuropsychological profiles suggesting a high risk of postsurgical cognitive impairment or patients with incongruent presurgical findings might benefit from the additional application of WT to provide them with as individualized information as possible. According to our findings, especially in patients with inconclusive HLL results from non-invasive imaging techniques, the WT could contribute to the individual prediction of potential cognitive decline after epilepsy surgery.

## Conclusions

Our findings confirm that Wada test (WT) results are of significant incremental value for the prediction of postsurgical change in verbal learning and verbal memory function. Thus, for a selected subgroup of patients with clear clinical indications, the WT remains an important part of the presurgical work-up and contributes to balancing out the risks and benefits of epilepsy surgery on an individual level.

In clinical routine and future research, the adaptation of outcome measures and the careful selection of adequate stimulus material for memory testing during the WT would facilitate the assessment of material-specific HML and, thereby, further improve the predictive value of WT results for postsurgical cognitive impairment.
